# Letter from Dr. Flint

**Published:** 1854-11

**Authors:** Austin Flint

**Affiliations:** Paris


### BUFFALO MEDICAL JOURNAL

AND

### MONTHLY REVIEW.

**VOL. 10.** NOVEMBER, 1854. **NO. 6.**

### ORIGINAL COMMUNICATIONS.

ART. I.—*Editorial Correspondence.* Letter from Prof. FLINT.

PARIS, June 26th, 1854.

DEAR DOCTOR: One of the most interesting of the public institutions that I have visited, is the veterinary school at Alfort, situated about six miles from Paris. There are three establishments in France under the direction of government, for veterinary instruction, and the treatment of the diseases of animals. That at Alfort is the largest. The two others are at Lyons and Toulouse. The institution at Alfort was founded in 1767, after a plan submitted by Bourgelot.

Leaving Paris at 7½ A. M., accompanied by my wife, and a female friend, we reached the *école* at 8½ o'clock. The morning consultation was then going on. Several horses were standing in the court. They were examined, in succession, by the clinical professor surrounded by a large number of *élèves*, and several minor surgical operations were performed. The morning visit to the horses confined in the stalls had been made before our arrival. After the consultation, an adjunct clinical professor, M. Reynal, a fine looking, intelligent, and courteous gentleman, very politely invited us into the reception room, and, ascertaining our wishes, directed one of the *élèves* to show us the entire establishment.

We visited first the stalls, which were well ventilated, clean, and in all respects comfortable. Distinct apartments are provided for horses which it is desirable should be kept isolated.

We were then conducted to the anatomical cabinet. In the center of the room are very fine skeletons, united and complete, of the dromedary, the horse, cow, hog, etc. Arranged in glass cases are skeletons of frogs, chickens, ducks, monkeys, and a number of other animals; also the disunited bones of the horse in profusion, all very neatly prepared and arranged. An adjoining room contains an artificial model of the horse, of the size of life, manufactured by *Azoux*, the muscular organs capable of being detached; several natural preparations of the entire subject, the muscles dried and varnished, on one of the animals thus prepared, the natural skeleton of a female being mounted *à la mode cavalier;* wet preparations showing the different periods of gestation, and a large collection of deformed and diseased bones. I noticed in this room a fine display in spirit of the glands of Peyer in the horse. The young *élève* who accompanied us stated that these glands, in the horse, are, normally, quite conspicuous. He also stated that they are found enlarged and ulcerated in certain cases in which the animals during life have appeared to be affected with a disease analogous to continued fever. This was to me new and highly interesting, suggesting the reflection that comparative pathology may perhaps prove as important in its bearings on our knowledge of human diseases, as the study of the structure of inferior animals has been of utility in the application of its results to the investigation of the organism of man.

In a third room was a large collection of concretions found in the stomach, intestines, and bladder of the horse and other animals. Also a series of excellent models, showing the appearance of the teeth at different ages; numerous injected vessels; a collection of minerals; different kinds of shoes for horses, etc., etc. In nicety of preparation and disposition, these several cabinets were not a whit inferior to the museums at the *école de médicine* of which I gave some account in my last letter.

We next visited the large botanical gardens connected with the institution. Here, among other classes of vegetable productions, were a great number of different species of grasses, each species being designated by its botanical title inscribed on porcelain.

From the gardens we walked through the park, beautifully ornamented with trees and shrubbery. Here were scattered some fifteen or twenty well dressed, fine looking young men, most of them reading. Owing to my engagements on week-days, I had selected Sunday morning for my visit to

Alfort, and our guide informed us that these young men were paying the penalty of having violated some of the rules of the institution, being obliged to remain at home, while their fellow-students had permission on this day to visit their friends.

The department appropriated to dogs was the next object of interest. In a small court, tied, were forty or fifty dogs, most of whom, the moment we entered, began to bark violently. Involuntarily we stepped back, until a scrutinizing glance showed that the animals, whose combined tones were frightfully ferocious, were perfectly secured. For the most part, they had been brought to the institution to be treated for *epizoa*. The animals seriously diseased were confined in kennels secured in front by iron rods. Here was a dog affected by *rabies*, the first that I had ever seen in which this disease undoubtedly existed. This dog was of the terrier species. It manifested violent irritation, barking, and making efforts to bite through the grating. There was no foaming at the mouth, but the teeth, on the contrary, were dry, and covered with sordes. The disease had been produced intentionally in this animal, by inoculating it with the saliva of a sheep affected with the disease. Our companion stated this curious fact, viz: the saliva from a rabid horse does not communicate the disease; but it is readily produced by inoculation with the saliva of a rabid sheep. At the access of the disease, convulsions are apt to occur, and death usually succeeds a comatose condition. I was surprised to learn that dogs live weeks, and even months, after the development of the affection. I was assured that there does not exist in dogs that inability to drink, or antipathy to fluids, which are presented in the human subject affected with hydrophobia.

An examination of the pharmaceutic department; the chemical laboratory and lecture-room; the apparatus for the illustration of physics; the general lecture-room, amphitheatre, dissecting room, and room for experimental operations, completed our tour of inspection. All these departments are as complete as possible. The arrangements for preparing and compounding medicines, and the chemical laboratory are really elegant, as well as complete. In the general lecture-room is a marble bust of Bourgelot, the founder of the institution.

The number of *élèves* at present connected with the institution is two hundred and fifty. They all live within the walls of the institution. A portion enter as gratuitous pupils, on the recommendation of the minister of commerce and agriculture; others pay a fee of about 75 dollars a year. Forty pupils are sent by the minister of war, and after leaving the institution, enter the cavalry service as veterinary surgeons.

They enter the school between the ages of seventeen and twenty-five, after an examination as to their ability to read and write correctly, and their acquaintance with certain elementary studies. They must also be practically conversant with the use of the forge. At the expiration of four years they are examined, and, if found competent, receive a diploma; if not competent, they are obliged to remain till the expiration of another year, or until the examination is satisfactory.

Horses and other animals requiring medical treatment are received at moderate charges, and gratuitonsly if the owners are poor. For a horse, 50 cents per day is the customary charge; and for a dog, 12 cents. A dog was admitted as I was leaving the institution, with, as the owner supposed, *ver solitaire.*

Horses unfitted by age, or other causes, for active service, are purchased for experimental observations, and to serve as subjects for the practice of surgical operations. Dissections are going on from November till April. Sheep and swine are also kept for experimental purposes.

The establishment embraces farriers' shops; and although it is not expected that the graduates of the school will be employed in shoeing horses, they must be qualified to know when it is properly done, to give directions as respects the operation, and to be able to do it themselves in cases of emergency.

The institution is placed under the immediate administration of a director, a steward, five professors, and three *chefs de service*, or adjunct professors. The departments of instruction belonging to the chairs severally are as follows: surgery and *clinique;* pathology; anatomy and physiology; chemistry and physics; agriculture and hygiene.

By means of this establishment and the two others already named, which, although less important, are conducted on the same plan, France provides her army, and the public, with a corps of scientific veterinary practitioners. With us, such establishments and such men are not to be found. Veteriinary surgery, as a science and art, so far as my knowledge extends, does not exist in America. A visit at Alfort, however, would, I think, impress you, as it did me, with the great importance of institutions for veterinary practice and education,: and I hope the deficiency in this respect in our own country will not much longer exist.

Very truly yours,

AUSTIN FLINT.

PARIS, June 28, 1854.

DEAR DOCTOR: In addition to the regular courses of instruction at the *école de médicine*, which I have specified in a former letter, and the clinical lectures by Trousseau, Velpeau, Nelaton, Cazenave, and others, at the hospitals, a great number of extra-official or private courses are still going on in Paris. The courses in which I have personally been much interested are the lectures by Prof. Bernard, on physiology, and the demonstrations in microscopy by Prof. Robin. Prof. Bernard's lectures are not extra-official or private, but they have no connection with the *école de médicine.* They are given at the *Sorbonne*, Prof. B. having recently been made a professor in the College of France.

The name of Claude Bernard is well known in the United States, not from his works, for I am not aware that any of the few publications that have as yet emanated from his pen have been translated, but mainly through letters by American physicians who have attended his lectures or followed his demonstrations in the laboratory. Among the letters referred to, those by Dr. Dalton, of New York, and Dr. Donaldson, of Baltimore, are particularly worthy of mention.

Prof. Bernard has for several years devoted his attention to *experimental* physiology, following in the footsteps of Majendie, with whom he was long associated as an assistant. The *élève* has already outstripped his master. Probably at this moment no one is more conspicuous, or more justly distinguished in the particular domain of science with which his name has become identified; and if his life be spared, inasmuch as he has hardly, as yet, arrived at middle age, much may be expected hereafter from his continued zeal and industry. The discovery of a new function of the liver is the most prominent among the fruits of his physiological labors. That this viscus forms sugar from the nutritious elements received by the portal vein, has been fully established by his numerous observations and experiments. He seems, moreover, to have proved, that the production of sugar by the liver is truly a *formative* process, that is to say, it is formed in the organ by a union of the simple elements, not merely by a conversion into sugar of compound substances which are capable, out of the body, of being thus transformed. He has found that animals restricted exclusively to a diet containing only nitrogenized elements, present the evidences of sugar in the liver. The saccharine matter produced in the liver is destroyed by respiration. It is only found in a state of health in the blood-vessels between the liver and lungs. Prof. B. has also demonstrated experimentally certain curious relations between the presence of sugar in the blood, and the condition of the nervous system—but these, together with other highly interesting but less striking discoveries, have been described in the letters of Drs. Dalton and Donaldson, and by others. I will only add, it is evident discoveries like that pertaining to the liver, are interesting and important, not alone in a point of view limited to physiological science. They are calculated, as are all striking discoveries in physiology, to exert not a little influence on pathology and therapeutics. Our notions with respect to *diabetes mellitus*, which have already experienced, during the last few years, several mutations in accordance with new light shed by the progress of physiology on the functions of the kidneys and of assimilation, must be materially modified in consequence of the grand discovery of Bernard.

In the course of lectures now in progress, Prof. B., up to the time I am writing, has been engaged mostly in treating of the nervous system. His style of lecturing is unaffected, easy and lucid. Like all the French lecturers that I have heard, he is fluent. This advantage, at least, is received by the system of *concouring*, since an aspirant who is notably deficient in ability to communicate his ideas, could hardly expect to triumph over his competitors. He uses the black-board frequently, and, whenever practicable, demonstrates the important points which he wishes to inculcate, by experiments on living animals. His course is numerously attended, his lecture-room being generally quite full; and his auditory appears to consist of a large number who are not medical students, judged by their age and general appearance.

A work by Prof. B. on experimental physiology is announced to appear shortly. It will be sought for with avidity by members of the medical profession in all countries.

Prof. B. has just been honored by an election to the Academy of Sciences, to fill the vacancy occasioned by the death of the eminent surgeon, Roux.

Dr. Charles Robin is professor *agregé* to the Faculty of medicine, his departments being general anatomy and microscopy. Within the last few years, several works relating to the subjects just mentioned, as well as numerous papers contributed to the Biological Society, have appeared from his pen. The elaborate treatise prepared by him in conjunction with Dr. Verneil, entitled *Anatomical Chemistry*, is a production of a very high order, embodying the results of original researches, much being new, and in a practical point of view, important. Prof. R. appears, by common consent, to hold the first place in Paris as a microscopical observer. He has been a laborious *worker*, as his publications testify, and his industry is still unremitting. He is not content with what he has already accomplished, and it is certain that his scientific mission is as yet far from being fulfilled, provided his constitution, (which seems to be somewhat delicate,) does not give way in his unceasing application to his favorite pursuits.

A private course of instruction in normal and pathological anatomy by Prof. R. is about concluded; and its completion will be immediately followed by the commencement of another course. Three evenings a week are devoted to these lectures, which are given in his private laboratory. In additon to his descriptive course, he has always two or three classes for practical demonstration with the microscope. Each class is limited to four persons. They meet at his laboratory three times a week, in the afternoon, and examine with the microscope specimens of healthy and morbid products. With the latter Prof. R. is abundantly supplied by the various hospital physicians and surgeons who wish to avail themselves of his opinion as to the pathological character of diseased parts coming under their observation. M. Robin is engaged in the composition of an elaborate and comprehensive treatise on general anatomy, normal and morbid, which will embrace a large number of plates illustrative of microscopical appearances, engraved from delineations by himself of observations in his own laboratory. Through the kindness of M. R., I have examined some of the drawings destined for this work. They are very beautiful, and will supply a want which is now much felt by those who wish to place themselves *au courant* with microscopical anatomy and pathology, but who cannot conveniently avail themselves of the demonstrations of a practical microscopist. Moreover, in matters of doubt and discussion, and many such pertain to this subject at present, views emanating from so distinguished an observer will be very acceptable. The work will require two years for its execution, and when completed, I am gratified to say that it is the intention of the author to pass a year in the United States.

The medical topic which has excited most interest in Paris during the last two months, is the treatment of deviations in position of the uterus. The subject was brought before the Academy of medicine by M. Valleix, who communicated some cases in support of the treatment of certain deviations by intra-uterine pessaries, as practiced by Prof. Simpson, of Edinburgh. M. Depaul, in behalf of a commission appointed by the Academy, made a long and elaborate report, taking strong ground against the introduction of foreign bodies within the uterus, and submitting a collection of cases in which serious results had followed this plan of treatment. A protracted discussion in the Academy has followed, different members expressing different views, but on the whole, M. Depaul's paper appears to be sustained. I

have already sent medical journals containing M. Depaul's report, and shall transmit subsequent numbers in which you will find the views of other distinguished speakers. A large portion of the columns of the various medical journals of Paris has been devoted to this topic for the last few weeks.

Having occasion to refer to medical journals, I am led to mention a melancholy event announced in the papers of this morning. Dr. Fabre, chief editor of one of the most popular and extensively circulated of the Parisian medical journals, day before yesterday, while walking in the street suddenly felt a sensation of faintness which led him to step into a *café.* He had hardly entered when he fell to the ground insensible, and expired in a short time. His death is attributed to sudden congestion of the brain. He was fifty-seven years of age. He was an able writer, and contributed largely to the French medical literature, in addition to his labors as a journalist.

The subject of hereditary syphilis is, at the present moment, under discussion at the meetings of the Academy of medicine. A paper was recently communicated by *M. Cullevier*, one of the surgeons of the hospital Lourcine, in which he broached the doctrine that the syphilitic affections developed shortly after birth are invariably derived from the mother, the father bearing no part in their transmission. The correctness of this doctrine is contested by *Ricord*, and other members of the Academy. The discussions are too protracted for me to give you even a *résumé*, but I shall supply you with the medical jouruals in which they are reported, that you may examine them at your leisure.

Epidemic cholera has existed in Paris to some extent during the three months that I have resided here. For the past two weeks, the number of cases at the hospitals have increased. The disease has been for the most part confined to the hospitals. There does not appear to have been evidence of an epidemic influence except that nearly all the hospitals have had, much of the time, a few cases in the wards, and in some (*La Charité*, especially) a number of cases have been developed within the wards. Nothing is said on the subject in the papers, and I presume few of the citizens of Paris, aside from those who frequent hospitals, are aware that the disease exists in the city. The medical journals are careful to publish bulletins showing the facts with respect to the progress of the disease. The *Gazette des Hopitaux* of the 24th inst., contains a list of the cases daily from the 15th inst., occurring at all the hospitals and hospices of Paris. The whole number for these seven days was 261. Of this number, 190 were received as cholera cases, and 71 were cases of persons attacked within the institutions. Of the 261 cases, 135 proved fatal, a proportion exceeding one half.

The same number of the journal just mentioned gives the whole number of cases that have occurred since the month of November last. The number is 2,596. Of this number, 1,370 died, and 214, at the time the enumeration was made, were under treatment.

It is apparent from these statistics, that the disease is not in a special degree amenable to French therapeutics. The cases being distributed among thirty or more institutions, I am unable to say what is the plan of treatment generally pursued. I will give, however, the course pursued in one of the wards at the hospital *St. Louis* in which I have been accustomed to make frequent visits, not directing, however, my attention to cholera cases, but to diseases of the skin, to which this hospital is specially devoted. My authority for the following statement of the treatment is the very intelligent *interne* of the ward referred to. As a special remedy, the physician of the ward places considerable reliance on the *Cannabis Indica*, the dose being, of the tincture, from 18 to 25 drops. But one dose only is usually given. Baths of mustard water are usually employed. Opium, in the form of the laudanum of Sydenham is prescribed in doses of 20 drops once during the 24 hours; and enemas containing 8 or 10 drops of laudanum morning and evening. Sinapisms to the epigastrium are relied upon to arrest vomiting. Ammonia is sometimes prescribed. My informant stated, as proof of the success of this practice, that five of twelve cases recovered.

Of French hospital practice in other affections than cholera, I may have something to say hereafter, if my leisure enables me to continue my correspondence.

With respect to hospitals, there is one fact (to which I think I have alluded in a former letter) at which I have been surprised. I refer to the custom of keeping full records of the cases from day to day. I had expected to find this course pursued at all the hospitals. This, however, is very far from being done. It is true that I have not yet visited all the hospitals, and I have directed my observations mainly to medical, as distinguished from the surgical wards; but so far as my present knowledge extends, I am able to mention but one instance in which I have witnessed a careful registration of clinical observations. This was at the hospital Lourcine, by M. Gosselin, one of the surgeons of that institution, whom I have mentioned in a previous letter. I state this simply as a fact which I had not anticipated, and which, if I mistake not, will appear surprising to many of my fellow-practitioners in America. Perhaps the idea that the Parisian hospital physicians are in the habit of recording the symptoms of diseases by the bedside has prevailed with us, from our acquaintance with the labors of Louis, and the high estimation placed on them on our side of the Atlantic. I do not mean to imply by the latter remark that these labors are not appreciated by his own countrymen, but it is certain that his system and example are not generally followed in this metropolis. Certain facts are noted at all the hospitals, and I presume required by government, viz: the age, residence, occupation, civil condition, disease, and result. In the wards of *M. Dubois*, at the hospital *des cliniques de la Faculté de médicine*, I noticed that the *billets de salle* affixed to the beds of the lying-in patients contained data for statistics as to the previous number of pregnancies, the state of health during gestation, the duration of labor, the presentation, interval between the delivery of the child and the placenta, etc. But usually, the only daily records made, so far as appears, relate to the remedies prescribed and the diet.*

Bedside instruction is given here by several of the hospital physicians by means of what are called *clinical conferences*. A certain number of *élèves* inscribe with the teacher, and meet him at the hospital at his morning visit. He calls upon each in rotation to examine cases newly admitted, to put to the patient all the questions he chooses, percuss, auscultate, etc., and then to give the diagnosis and appropriate treatment. I have witnessed these clincal conferences at the hospital *St. Antoine* by M. Aran, at *La Charité*, by M. Piorry, and at *La Pitié*, by M. Valleix. With those at the hospital last named, I was especially pleased. After the pupil has finished his examination, M. Valleix pointed out omissions in the interrogations or explorations, reviewed the facts, and, if necessary, corrected the conclusions at which the pupil had arrived. This is eminently practical instruction, and is certainly a most useful exercise for the student.

It is, however, attended with some inconveniences. The student is sometimes so timid and embarrassed that he requires to be continually pushed on; in some instances, too, his incompetency is so great, that he has to be corrected at every step, and, again, sometimes, his egotism and assurance lead him to ask a great multiplicity of needless questions, and to prolong the examination to a tedious extent. An improvement, as it seems to me, would be to require an examination to be made by himself at his leis-

* The following is a list of the several headings under which the daily directions are entered by the internes who accompany the physician, and afterward signed by the latter:

1. *Tisanes.* 2. *Medicaments internes.* 3. *Medieaments externes.* 4. *Bouillions.* 5. *Potages du riz ou vermicelle,* (a) *au gras,* (b) *au lait.* 6. *Soupes au pain,* (a) *graisse,* (b) *maigre.* 7. *Aliments solides,* (a) 1 *portion,* (b) 2 *portions,* (c) 3 *portions,* (d) 4 *porions.* 8. *Boissons alimentaires,* (a) *vin,* (b) *lait.*

ure, and a written history, with the conclusions as respects diagnosis and treatment, to be presented, read, commented on and discussed at the morning visit. At a clinical conference by M. Valleix at which I was present on the day I am writing, two hours were consumed by the examination of two cases; and the examination of other patients was of necessity considerably abridged.

From the number and size of the hospitals in Paris, it would be inferred that the aggregate receptions in the course of a year must amount to many thousands. I have been informed that one-third of all the deaths which take place in Paris, occur at public institutions. In 1850, there were treated at the different hospitals under the control of "the general administration of public assistance," 88,949 patients. This is exclusive of the cases at the *hospices,* or alms-houses, and the *maisons de retraite.* If the latter were to be included, the number would be increased by 11,973.

The rate of mortality has steadily diminished during the last forty years. In 1805, the deaths were in the proportion of 1 to 5 at the general hospitals, and of 1 to 15 at the special hospitals. In 1850, 1 of 11 patients in the general hospitals died, and at the special hospitals, 1 of 17. The intermediate years show a progressive gradation in the proportion of recoveries, so that the above contrast is not to be explained by greater tendencies in diseases to a fatal issue during the two periods stated. It is a fair inference from these facts, that in hygienic conditions, or in the management of diseases, or in both combined, there has been continued improvement. That there is room for farther improvement, which will reduce still more the rate of mortality, is undoubtedly true; but so far as concerns the general administration of the hospitals, the provisions for ventilation, cleanliness, nursing, etc., I must say I have been agreeably disappointed. I had not expected to find them so free from obvious unfavorable circumstances.

As you perceive, my dear sir, I am writing of different matters as they suggest themselves to my mind, and my letter will, therefore embrace a heterogeneous collection of topics. Reverting to cholera, a French gentleman deceased some time since, left a bequest of a considerable sum (I think ten thousand dollars) to be awarded by the Academy of Sciences to the discoverer of a cure for that disease. The hope of getting this reward has stimulated some Americans, not of the medical profession, to transmit certain recipes which they believe to possess unfailing specific virtues. Some letters enclosing these recipes were read at a meeting of the American Society, and as specimens of chirography and orthography, as well as on account of the singular character of the remedies and combinations communicated, they were sufficiently amusing. The evidence offered in behalf of the efficacy of the prescriptions was that they had proved successful in the persons of the applicants for the reward.

Passing from the cholera to the *jardin des plantes*, in a late letter in which I gave some account of the *Musée Orfila*, I mentioned that I had not then visited the cabinets of the former place. I have recently passed a portion of several days there, examining the collections in comparative anatomy, botany and geology, and zoology. To give any idea of the details embraced in these collections by a description within the limits of a letter, would be impossible. I will only say that to pass a week in the *jardin des plantes* seems to me, of itself, almost to repay for a voyage across the Atlantic. The assemblage of skeletons in the cabinet of comparative anatomy is immense. In this respect, the Orfila Museum cannot be compared with it. In preparations of the soft parts, however, the Orfila Museum is superior.

Courses of lectures on chemistry, zoology, botany, physics, comparative anatomy, mineralogy, etc., are in progress at the *jardin des plantes*, which, like the lectures at other public institutions, are free. At these lectures, ladies are admitted.

I desire to retract certain expressions in my first letter relative to the climate of Paris. For the first three weeks after my arrival, in April, the weather was delightful. During this time no rain fell, and so much danger to the crops was apprehended were the drought to continue, that prayers for rain were ordered by the Archbishop of Paris. For the last two months, it is not much exaggeration to say that it has rained continuously. Certainly there have not been during this period, a half dozen days of fine weather, and never have there been two days without rain in succession. Moreover, most of the time, fires and overcoats have been essential to comfort. Instead, therefore, of saying that even the elements of nature appear to have combined to render Paris an attractive city, it seems to me now nearer the truth to attribute its charms to the triumph of art over the natural disadvantages of climate. It is said here that such an unpleasant season was never before known, but it is well understood this is a very common method of speaking of unfavorable weather.

Very truly, yours,

AUSTIN FLINT.

